# The Overlooked Threat of Malnutrition: A Point Prevalence Study Based on NRS-2002 Screening in a Tertiary Care Hospital

**DOI:** 10.3390/jcm14113976

**Published:** 2025-06-05

**Authors:** Ekmel Burak Özşenel, Güldan Kahveci, Yıldız Pekcioğlu, Beytullah Güner, Sema Basat

**Affiliations:** 1 Department of Internal Medicine, University of Health Sciences, Ümraniye Education and Research Hospital, Istanbul 34764, Turkey; yildizpekcioglu@gmail.com (Y.P.); beytullah17guner@gmail.com (B.G.); semaucak@hotmail.com (S.B.); 2 Nuritional Nursing, Department of Internal Medicine, University of Health Sciences, Ümraniye Education and Research Hospital, Istanbul 34764, Turkey; nurse.guldan@gmail.com

**Keywords:** malnutrition, nutritional support, point prevalence

## Abstract

**Background:** Malnutrition is increasingly prevalent due to rising life expectancy, oncological cases, and chronic diseases. Early detection is crucial for rehabilitation, complication prevention, and cost reduction. However, nutritional support is often suboptimal. This study aimed to determine malnutrition prevalence and nutritional support status within our hospital. **Methods:** A point prevalence study was conducted in adult inpatient clinics (excluding pediatrics, infectious diseases, and intensive care) by a 12-member team following ethical approval. NRS-2002 scores, arm/calf circumferences, BMI, and laboratory data (albumin, leukocytes, lymphocytes, neutrophils, hemoglobin, CRP, creatinine) were assessed. Enteral and parenteral nutrition treatments were recorded. Patients with NRS-2002 scores ≥ 3 were classified as at risk of malnutrition. **Results:** Among 178 patients, 24.7% were at risk of malnutrition. Surgical clinics had a higher malnutrition risk (32.3%) than internal medicine clinics (20.3%). Only 27.1% of at-risk patients received nutritional support (surgical: 19%, internal medicine: 44%). Patients at risk of malnutrition exhibited significantly lower arm circumference (*p*: 0.000), calf circumference (*p*: 0.002), lymphocyte counts (*p*: 0.000), hemoglobin (*p*: 0.018), albumin (*p*: 0.001), and BMI (*p*: 0.038), as well as significantly higher age (*p*: 0.000) and CRP levels (*p*: 0.000). **Conclusions:** Nutritional support remains inadequate despite increased attention to malnutrition. Intensified nutrition education, particularly in surgical inpatient clinics, is needed to improve patient rehabilitation and outcomes.

## 1. Introduction

Malnutrition is a complex and multifaceted condition. It is described variably across different health organizations. The World Health Organization defines malnutrition as a state resulting from both insufficient and excessive nutrient intake [1]. In contrast, the European Society for Clinical Nutrition and Metabolism (ESPEN) focuses specifically on nutritional deficiencies and their clinical consequences. Malnutrition is broadly characterized by the inability of the body to acquire essential nutrients in adequate quantities, resulting in metabolic, functional, and biochemical disturbances. These disruptions can manifest as tissue wasting, muscle loss, cellular dysfunction, and immune system impairment [2].

Multiple associations provide differing definitions and screening tools for malnutrition, incorporating subjective assessments and objective measurements such as anthropometry and biochemical parameters [3,4,5]. Despite this variation, the most widely accepted definition centers on the failure to meet the body’s energy and nutrient demands. Risk of malnutrition can develop under a range of conditions, particularly those that elevate nutritional needs. These include physiological states like pregnancy and growth, as well as medical conditions such as cancer, chronic kidney disease, gastrointestinal disorders, and other chronic illnesses. Additionally, acute stressors like surgery, trauma, burns, and infections may also exacerbate nutritional demands, thereby increasing malnutrition risk.

Malnutrition is more frequently observed in the elderly and hospitalized patients, although prevalence rates vary by geographic location. In Türkiye, studies have reported malnutrition prevalence values between 13% and 35% in tertiary care settings [6,7]. A global meta-analysis identified substantial regional variability, with malnutrition prevalence among elderly individuals ranging from 0.8% in Northern Europe to 24.6% in Southeast Asia [8]. Furthermore, clinical units with specialties such as geriatrics, oncology, and gastroenterology often report the highest rates of malnutrition among inpatients [9,10,11].

The clinical and economic consequences of malnutrition are substantial. Among hospitalized elderly patients and those with chronic illnesses, malnutrition is associated with delayed wound healing, an increased risk of pressure ulcers, infections, and sepsis. These complications result in longer hospital stays and higher healthcare expenditures. One study revealed that malnourished patients incurred hospital costs that were 19% to 29% higher than those of adequately nourished patients, amounting to an average increase of €416 to €617 [12]. Another study found that patients consuming only 25% of their required daily energy intake stayed in hospital approximately two days longer than those consuming 50% [13]. Similarly, inadequate dietary intake was linked to higher rates of healthcare-associated infections when patients failed to meet at least 70% of their predicted energy needs [14].

The timely evaluation of malnutrition risk during hospital admission, combined with appropriate nutritional interventions for those identified at risk, is crucial. This approach not only enhances recovery but also reduces complications, mortality, and overall healthcare burden. A proactive nutritional strategy at the outset of hospitalization can thus contribute significantly to patient outcomes [15].

Despite the growing awareness of malnutrition and the availability of validated screening tools, nutritional support remains insufficient in clinical practice. It is hypothesized that this is largely due to the heavy workloads of healthcare professionals and their tendency to prioritize disease-specific interventions over comprehensive nutritional care. As a result, patients at risk of malnutrition may not receive the necessary support. In response to these gaps, the present study was designed with two main objectives: (1) to determine the prevalence of malnutrition among inpatients in a tertiary care hospital, and (2) to assess the nutritional support status of patients identified to be at risk.

## 2. Materials and Methods

Ethical approval for the study was granted by the Ethics Committee of Ümraniye Training and Research Hospital on 8 October 2020 (Approval No: B.10.1.TKH.4.34.H.GP.0.01/329). The present study was designed as a ‘point prevalence study’ and conducted on 19 October 2020 in our tertiary care hospital, with a team comprising 12 individuals, including 10 physicians, a dietitian, and a nurse who were members of the nutritional support team. The sample size was calculated using the formula for a single proportion estimation. With a 95% confidence level and a 5% margin of error, the required sample size was found to be 169.

Before starting the study, the entire team was apprised of the utilization of a tape measure for the standardization of anthropometric measurements. Furthermore, a practical application was conducted prior to the screening, enabling all participants to undertake a standardized assessment while applying the NRS-2002 test. This approach was adopted to mitigate the potential for errors in assessment.

Patients hospitalized in the pediatric, infectious diseases and intensive care units were excluded. Patients who had undergone an extremity amputation were excluded from the study to avoid potential errors in body mass index and arm–calf circumference measurements. The study included patients aged 18 years and over. All participants provided written informed consent prior to inclusion in the study.

Each member of the screening team was given a standard tape measure to take height, arm circumference, and calf circumference measurements of the patients. Patients who could not stand up had their height measured in bed. Patients who could stand up had their weight determined using scales of the same brand used in all hospital inpatient clinics. Bedridden patients were moved to a bed with scales located in one area of the hospital, where their weight was measured. The body mass indexes of the patients were also calculated on the basis of height–weight measurements. Also, demographic information about the patients and the values of albumin (g/dL), leukocytes (10^3^/µL), lymphocytes (10^3^/µL), neutrophils (10^3^/µL), hemoglobin (g/dL), CRP (mg/L), and creatinine (mg/dL), assessed via the tests requested during hospitalization, were obtained from the hospital automation system and recorded. In order to clearly reflect the patient’s condition, it was ensured that the above laboratory tests recorded for all patients included in the study were performed on the day of screening. In the laboratory, hemogram is studied in Mindray BC6800 (Shenzhen Mindray Bio-Medical Electronics Co., Shenzhen, China) device, and CRP, creatinine, albumin are studied in Roche Cobas 8000 (601 series) (Roche Diagnostics, Mannheim, Germany) device. In addition, we found out whether patients were receiving enteral nutrition (tube feeding), oral nutritional supplements, or parenteral nutrition from the inpatient clinic nurses and recorded. Furthermore, the NRS-2002 score, a screening parameter for malnutrition, was calculated at the bedside. Patients with a score of 3 or more were considered at risk of malnutrition [3].

**Nutritional Risk Screening Test-2002 (NRS-2002):**

The Nutritional Risk Screening 2002 (NRS-2002), introduced by Kondrup et al., was designed to identify malnutrition risk among hospitalized individuals [3]. This scoring system enables the quantitative determination of mild, moderate, or severe malnutrition risk. The test comprises two primary components. The first part evaluates the deterioration in the nutritional status of the individual, while the second part analyses the severity of the disease. Each section of the test is assigned a score ranging from 0 to 3, with an additional 1 point given for patients aged 70 years and over. A total score of three or more indicates a risk of malnutrition and therefore requires the implementation of a nutritional support plan. The complete test can be seen in Table 1.

**Statistical analysis:**

Statistical analyses were conducted using the IBM SPSS 26.0 (Statistical Package for the Social Sciences) program (SPSS Inc., Chicago, IL, USA). Descriptive statistical methods, including mean, standard deviation, median, frequency, percentage, minimum, and maximum, were performed to evaluate the study data. The distribution of data was tested using the Kolmogorov–Smirnov test. The parametric two-sample independent *t*-test was used for normally distributed data and the Mann–Whitney U test, a non-parametric test, was used for non-normally distributed data. The chi-squared test and Fisher’s exact test were used to evaluate categorical data. The significance level was set at *p* < 0.05 for all values.

## 3. Results

The study population consisted of 178 patients (89 males and 89 females). A total of 48 patients (26 males and 22 females) were identified as being at risk of malnutrition. When the entire cohort of inpatients was considered, we found that 24.7% of patients had malnutrition risk. As shown in Table 2, the rate of patients at risk of malnutrition was 32.3% in surgical inpatient clinics, including general surgery, ear-nose-throat, gynecology, neurosurgery, orthopedics, and urology. Conversely, the rate was 20.3% in internal inpatient clinics, including internal medicine, endocrinology, gastroenterology, nephrology, neurology, and cardiology.

When all patients with an NRS-2002 score of 3 or above were considered, it was found that only 27.1% received enteral or parenteral nutrition support (Table 3). When surgical and internal inpatient clinics were evaluated separately in terms of nutritional support, it was found that 19% of patients at risk of malnutrition in surgical inpatient clinics and 44% in internal inpatient clinics received nutritional support (Table 3). In addition, malnutrition risk rates of all inpatient clinics are shown in detail in Table 4.

When all the data of the two groups were compared, it was found that the arm and calf circumference measurements, lymphocyte counts, BMI values, albumin, and hemoglobin values of the group with NRS-2002 score 3 and above were statistically significantly lower than those without malnutrition risk (Table 5). Furthermore, the mean age, number of hospitalization days, and CRP values of the group with NRS-2002 scores of 3 and above were found to be statistically significantly higher than in the group without malnutrition risk (Table 5).

## 4. Discussion

The primary aim of this study was to assess the prevalence of patients at risk of malnutrition within a tertiary care hospital and to determine the proportion of these patients who received appropriate nutritional support.

Malnutrition is increasingly prevalent due to a combination of prolonged life expectancy and the rising incidence of chronic diseases among the elderly population. While healthcare policies have established guidelines to identify malnourished patients and ensure supportive nutritional interventions, the practical implementation of these protocols remains suboptimal. This gap may stem from insufficient awareness among healthcare professionals, inadequate training, or systemic inefficiencies that hinder the integration of nutritional care into routine clinical workflows.

Our study revealed that 24.7% of the hospitalized patients were at risk of malnutrition, a figure that aligns with those reported in similar inpatient studies across various healthcare settings [6,7].

Perhaps one of the most concerning results of our study is that only 27.1% of patients identified as being at risk of malnutrition received a documented nutritional intervention. In effect, nearly three-quarters of malnourished patients were overlooked. Likewise, many studies on malnutrition awareness, in which different healthcare professionals were evaluated, concluded that patients could not receive complete nutritional support treatment due to reasons such as a lack of awareness, inadequate education, or insufficient cooperation between units [16,17,18,19].

An interdepartmental comparison within our study revealed that surgical inpatient clinics exhibited a higher risk of malnutrition compared to internal medicine wards. More notably, the proportion of patients receiving nutritional support was lower in surgical departments, despite the higher risk. Although these differences did not reach statistical significance, they suggest the existence of a concerning trend that warrants further investigation through larger, multicenter studies. These findings also call attention to potential disparities in training and awareness among different clinical specialties.

Based on our observations, we believe that current in-hospital nutrition education programs, as administered by the Ministry of Health, may not adequately address the needs of specific clinical settings. Rather than employing generalized online or centralized in-service training, we advocate for department-specific education programs. Tailored, face-to-face training sessions within high-risk departments may enhance the recognition and management of malnutrition. Additionally, we recommend routine audits to identify departments with either a high prevalence of malnutrition or low rates of nutritional intervention, allowing targeted education and quality improvement initiatives.

The present study demonstrated that the mean age of patients with malnutrition risk was significantly higher than that of the group without malnutrition risk. In addition, the extant literature contains studies that demonstrate the existence of a high risk of malnutrition in the geriatric population, as evidenced by the parallel with our own research [20,21,22]. A large-scale study undertaken by Yan Liu et al. in a cohort of inpatients revealed that the prevalence of malnutrition was higher in male subjects than in female subjects [23]. However, the present study did not identify any differences in the risk of malnutrition between sexes.

In their study Chites et al. demonstrated that the presence of malnutrition resulted in a prolonged duration of hospitalization [24]. Furthermore, Sganzerla et al. demonstrated that even in cases of obesity, the duration of hospitalization was prolonged in the presence of malnutrition, and readmission rates increased following discharge [25]. In light of the fact that the present study was a point prevalence study, and given that the duration of hospitalization following the study day was not included, it is not possible to make a definitive interpretation that malnutrition prolongs the duration of hospitalization. Consequently, the increased number of hospitalization days among the malnourished group, although noteworthy, is not considered a significant result among the parameters noted on the study day.

The present study found that albumin values in the malnourished group were significantly lower than in the non-malnourished group. Although low albumin levels are generally associated with malnutrition, we hypothesize that the observed decrease in albumin may be a consequence of negative acute-phase reactant status, as indicated by elevated C-reactive protein (CRP) levels in the malnourished group. Furthermore, the lymphocyte count is widely accepted as an additional parameter used to indicate malnutrition [26,27]. When this parameter was evaluated in the present study, it was found that the lymphocyte count was significantly lower in the group at risk of malnutrition. It is believed that, in comparison to low albumin, lymphopenia can be accepted as an indicator of malnutrition that is unaffected by elevated CRP.

This study had several limitations. The study was conducted during the COVID-19 pandemic. Therefore, although there were 300 beds available in the inpatient clinics included in the study, only 178 patients were able to participate in the study. While our results are largely consistent with the existing literature, a more comprehensive analysis involving the full inpatient capacity could offer more robust and generalizable conclusions. Moreover, we believe that some of our statistically non-significant but numerically meaningful findings—such as interdepartmental differences in nutritional support—could reach significance with a larger patient sample.

## 5. Conclusions

The findings of this point prevalence study demonstrated that the prevalence of malnutrition at the study center was in line with previously published data. However, the rate of nutritional support among at-risk patients was notably low. Especially in surgical inpatient clinics, patients were more likely to be malnourished and less likely to receive nutritional support. It is believed that the implementation of capacity building of health workers in the identification of malnutrition and targeted nutrition support, with a particular focus on surgical inpatient clinics, will result in a marked improvement in outcomes.

## Figures and Tables

**Table 1:** Nutritional Risk Screening 2002.

1. Initial Screening				Yes	No
1. Is the BMI < 20?					
2. Has the patient lost weight within last 3 months?					
3. Has the patient had a reduced dietary intake in the last week?					
4. Is the patient severely ill?					
If the answer to any question is “yes” final screening is performed					
If the answer is “no” to to all questions, patient should be screened once a week, if there is a major operation, a nutritional plan should be made					
**2. Final Screening**					
Impaired nutritional status		Severity of disease			
Abcent Score 0	Normal nutritional status	Abcent Score 0	Normal nutritional requirements		
Mild Score 1	>5% weight loss in 3 months or food intake below 50–75% of normal requirement preceding week	Mild Score 1	Hip fracture chronic patients, in particular with acute complications: Cirrhosis, COPD, chronic haemodialysis, diabetes, oncology		
Moderate Score 2	Weight loss > 5% in 2 months or BMI 18.5–20.5 + impaired general condition or food intake 25–50% of normal requirement preceding week	Moderate Score 2	Major abdominal surgery, stroke, severe pneumonia, haematological malignancy		
Severe Score 3	Weight loss > 5% in 1 month (>15% in 3 months) or BMI < 18.5 + general impairment or food intake 0–25% of normal requirement preceding week	Severe Score 3	Head trauma, bone marrow transplantation, intensive care patients (APACHE > 10)		
Age	If ≥ 70, add 1 to the total score				
Score ≥ 3 The patient is at nutritional risk and a nutritional plan is initiated.					
Score < 3 Should be screened once a week, if there is a major operation, a nutritional plan should be made					

**Table 2:** Sex and clinic characteristics of patients in terms of NRS-2002.

		NRS-2002						
		<3		≥3		Total		
		N	%	N	%	N	%	*p* *
Sex	Male	63	70.8	26	29.2	89	100	0.499
	Female	67	75.2	22	24.8	89	100	
Inpatient Clinic	Surgical	67	67.7	32	32.3	99	100	0.071
	Internal	63	79.7	16	20.3	79	100	

* Pearson chi-square.

**Table 3:** Nutritional support status of patients with NRS-2002 score ≥ 3.

	Nutritional Support (−)		Nutritional Support (+)				
NRS-2002 ≥ 3	N	%	N	%	N	%	*p* **
Surgical inpatient clinics	26	81.3	6	18.7	32	100	0.09
Internal inpatient clinics	9	56.3	7	43.7	16	100	
Total	35	72.9	13	27.1	48	100	

** Fisher’s exact test. Nutritional support (−): patient is not receiving enteral or parenteral nutrition support. Nutritional support (+): patient is receiving enteral or parenteral nutrition support or both.

**Table 4:** Distribution of patients with and without malnutrition risk by the inpatient clinics.

Inpatient Clinics	NRS-2002 < 3		NRS-2002 ≥ 3		Total	
	N	%	N	%	N	%
Internal medicine	10	7.7	12	25	22	12.4
Endocrinology	5	3.8	0	0	5	2.8
Gastroenterology	17	13.1	0	0	17	9.6
General surgery	11	8.5	22	45.8	33	18.5
Cardiology	11	8.5	0	0	11	6.2
Otorhinolaryngology	8	6.2	1	2.1	9	5.1
Gynecology	15	11.5	0	0	15	8.4
Nephrology	12	9.2	1	2.1	13	7.3
Neurology	8	6.2	3	6.3	11	6.2
Neurochirurgy	7	5.4	4	8.3	11	6.2
Orthopedics	16	12.3	1	2.1	17	9.6
Urology	10	7.7	4	8.3	14	7.9
Total	130	100	48	100	178	100

**Table 5:** Comparison of patients with NRS-2002 score < 3 and ≥3 in terms of laboratory tests and anthropometric measurements.

	NRS-2002 < 3		NRS-2002 ≥ 3		
	Mdn	IQR	Mdn	IQR	*p*
Age	56	27.3	65	31.3	0.001 *
Days of hospitalization	3	3	6	10.25	0.000 **
BMI	28.8	6.98	26.1	6.45	0.038 *
Arm circumference (>22 cm)	30	6	26	6	0.000 **
Calf circumference (>31 cm)	35	5	33	6	0.002 *
Crp (mg/L) (0–5)	13	49.08	83.9	169.67	0.000 **
Albumin (g/dL) (3.5–5.2)	3.8	0.98	3.25	1.45	0.001 **
Creatinine (mg/dL) (0.7–1.2)	0.8	0.56	0.78	0.69	0.950 **
Wbc (10^3^/uL) (4–10)	8.7	4.55	7.77	4.41	0.363 **
Neutrophils (10^3^/uL) (2–7)	6.1	4.28	6.24	4.1	0.579 **
Lymphocytes (10^3^/uL) (0.8–4)	1.89	1.22	1.06	0.93	**0.000** **
Hemoglobin (g/dL) (12–16)	11.5	4	10.5	3	**0.018** *

Mdn: median. IQR: interquartile range. * independent *t*-test. ** Mann–Whitney U test.

## Data Availability

The authors declare that the data of this study are stored and can be accessed by contacting the corresponding author (Ekmel Burak ÖZŞENEL) if necessary. The study data are not publicly available due to personal data protection laws in Türkiye.
